# Neutron scattering observation of quasi-free rotations of water confined in carbon nanotubes

**DOI:** 10.1038/srep45021

**Published:** 2017-03-22

**Authors:** G. Briganti, G. Rogati, A. Parmentier, M. Maccarini, F. De Luca

**Affiliations:** 1 Sapienza Univ. of Rome, Dept. of Physics, Rome, 00185, Italy; 2 Tor Vergata Univ. of Rome and NAST Center, Dept. of Physics, Rome, 00133, Italy; 3 Grenoble Alpes Univ., Lab. TIMC/IMAG UMR CNRS 5525, La Tronche, 38700, France

## Abstract

The translational and orientational dynamics of water in carbon nanotubes has been studied by quasi-elastic neutron scattering from 300 down to 10 K. Results show that, reducing temperature below 200 K, part of this water behaves as a quasi-free rotor, that is, the orientational energy of such molecules becomes comparable to the rotational energy of water in the gas phase. This novel and unique dynamic behavior is related to the appearance of water molecules characterized by a coordination number of about two, which is promoted by sub-nanometer axial confinement. This peculiar molecular arrangement allows water to show an active rotational dynamics even at temperatures as low as 10 K. The translational mobility shows a behavior compatible with the rotational one.

Nanoscale confinement is probably the dominant condition in which water plays its role in biology, chemistry, geology or material science. Confinement affects the hydrogen-bond structural dynamics of water modifying its properties, therefore giving it features that make water functional to specific roles. In the last decades the effect of the confining scale and geometry has become a very important area of research, especially for those issues concerning the so-called supercooled liquid state, which has proven of great importance from a biological point of view^1^.

Among all confining geometries, the physical properties of water in a quasi-one-dimensional confinement have attracted particular interest, mostly because of the transport properties displayed by water under this condition^2^,^3^,^4^,^5^. The axial confinement on a sub-nanometer scale may reduce the coordination number of water to a value as low as about 2, with bonds at approximately 180° from each other, therefore deeply modifying the rotational and translational dynamics of molecules with respect to the bulk^6^,^7^,^8^,^9^.

The issues inherent to this molecular arrangement are of both fundamental and applicative interest, since they are part of the general topic concerning molecules’ organization to comply with a specific confining geometry, while, at same time, they give information about the dynamics of water permeating biological trans-membrane channels.

Since rotational and translational entropies of molecules increase to compensate for the reduction of bond energy per molecule^3^,^4^,^5^,^10^, rotational and translational diffusion of water in the axial hydrogen-bond arrangement, which makes one of water’s hydrogens unconstrained by the hydrogen bond, should show features of an unusual gas-like dynamics, that is, a net increase of mobility should manifest with respect to the bulk at fixed temperature. These atypical dynamic features make the genesis of the single-file of water molecules experimentally testable. Moreover, the observation of these special dynamic conditions would be a confirmation of the quasi-linear hydrogen-bond configuration adopted by water to match axial confinement.

Carbon nanotubes are effective tools for creating quasi-one-dimensional molecular confinement. Water entrapped in single-walled carbon nanotubes (SWCNTs) is the most studied model of axial confinement, since it also involves applicative issues, which range from ion filtering^11^ to the construction of molecular nanovalves^12^. SWCNTs are susceptible of accurate characterization in terms of diameter and (*n, m*) chiral indices^13^. Water in SWCNTs has been mainly studied by molecular-dynamics (MD) (see review^14^ and references therein), and, to a lesser extent, by experimental microscopy^15^ and spectroscopic methods^6^,^9^,^16^,^17^,^18^,^19^,^20^,^21^,^22^,^23^,^24^.

In this paper we study the translational and rotational diffusion of water trapped in up-to-50-*μ*m-long (10, 10) SWCNTs of diameter *d* = (1.4 ± 0.1) nm by quasi-elastic neutron scattering (QENS), in the temperature interval from 300 to 10 K, with the purpose of observing the formation of the single-file water chain and nearly free-rotation of molecules. QENS is particularly suitable for this task as it essentially probes the hydrogen dynamics through the incoherent structure factor of hydrogen around the elastic region, giving information on the geometry of the diffusing system and the time scale of diffusion, ps in our case^25^. The onset of sub-nanometer axial confinement in the (10, 10) SWCNTs should occur below 200 K, after the formation of a square-ice sheet rolled to form a channel coaxial to the nanotube^6^,^16^ (Fig. 1). Therefore any peculiarity of water’s roto-translational dynamics below this temperature may become the signature of the emergence of the single-file structure and dynamics.

## Results

In order to fit QENS spectra, the decoupling between vibrational, translational and orientational motions has to be assumed^26^,^27^,^28^. The Debye-Waller factor, which describes the attenuation of the scattering intensity due to the thermal motion of water molecules, has the form ~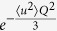, 〈*u*^2^〉 being the mean square displacement of hydrogen from the equilibrium position and *Q* the momentum transfer. Considering that the O–H length in a water molecule is about 1 Å, the Debye-Waller factor is essentially unity in the entire investigated *Q*-range (0.38 ≤ *Q* ≤ 2.0 Å^−1^), since 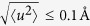
29. Indeed, tests made on our QENS spectra at all temperatures have, in fact, confirmed that either the inclusion or the exclusion of this factor does not actually alter the fits of the experimental data.

The scattering function is composed by two Lorentzian functions, which are related to the translational and rotational diffusion of molecules, plus an elastic term, which takes into account the immobile hydrogen atoms, that is, hydrogens possibly locked into ice structures^30^,^31^. The QENS scattering function relies on the so-called decoupling approximation, which means that the translational and rotational degrees of freedom are decoupled from each other. Yet, in condensed water translations and rotations are basically coupled by the hydrogen-bond cage effect, so that the decoupling approximation has been predicted to break down at large *Q*’s at low temperature^27^.

In our system, even at the largest momentum available at IN6, the decoupling approximation should work, within a negligible error, down to about 190 K^29^. However, with our QENS measurements covering the majority of the sub-nanometer-confinement temperature region - which, as mentioned above, is expected to extend below about 200 K - the decoupling approximation should apply at temperatures below about 190 K as well. Indeed, water arranged as a single-file structure has features that could favor the decoupling also at temperatures well below 190 K. Precisely, the effective molecular reorientation axis in the water single-file configuration is necessarily close to the hydrogen-bond direction, therefore the bond cage is expected to get poorly strained by rotational diffusion; rotational diffusion might even occur without breaking any bond. A similar consideration applies to translation, which occurs nearly along the bond axis, therefore without significant distortion of the bond cage symmetry.

All that said, the QENS incoherent scattering cross function can be written as the following convolution product


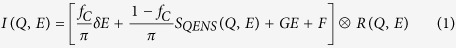


where


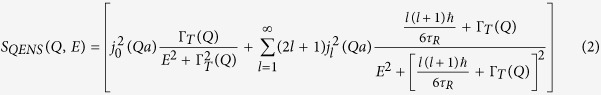


In Eq. 1 *R(Q, E*) is the experimental resolution function, *f*_*C*_ is the fraction of immobile protons^24^,^30^,^31^ and *GE* + *F* takes the inelastic background into account, with *G* and *F* constants. The *f*_*C*_ term returns the fraction of immobile water that gets formed, providing an indication about the temperature-dependent single-file host-channel formation process.

In Eq. 2 *τ*_*R*_ is the rotational relaxation time, Γ_*T*_ is the translational half-width at half maximum of the translational Lorentzian, *a* is the radius of molecular rotation, which has been fixed to the O–H distance, namely 0.98 Å, and *j*_1_(*Qa*) is the l^*st*^-order spherical Bessel function^26^,^28^. *S*_*QENS*_ is expressed through the Sears expansion^32^. Strictly speaking, Sears’ model for the water molecule refers to the motion of hydrogen atoms around the molecular center of mass, i.e., around the oxygen atom. Because of the confining geometry, orientational motion in fact occurs around an axis passing through the oxygen atom, which is almost coincident with the channel axis. Considering the *Q* values spanned in this experiment, the terms with *l* > 1 can be neglected^26^, thus only the *l* = 1 term is kept in Eq. 2.

It has been shown that the translational diffusion of water under this confining condition is Fickian, also when the channel diameter gets reduced to less than two times the equivalent hard-sphere molecular diameter, if the formation of molecular clusters is taken into account^33^,^34^,^35^. In this case, the relation that connects the translational diffusion coefficient *D*_*T*_ to the Lorentzian half-width is given by 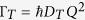.

In Figs 2 and 3 some scattering spectra are shown as examples of our fit procedure. The instrumental resolution function was measured on the dry sample, namely on empty SWCNTs, at each operational temperature.

### Rotational dynamics

The temperature dependence of the *τ*_*R*_ rotational relaxation time, obtained from the fit to Eq. 1, is reported in Fig. 4.

These rotational relaxation times match the correct order of magnitude^10^,^36^,^37^,^38^. The *τ*_*R*_-vs.-*T* behavior of Fig. 4 is fully compatible with the formation of the single file of water molecules. Indeed, from 300 down to 250 K, *τ*_*R*_ increases as expected for a thermally-activated motion. If one supposes that rotational diffusion is driven by an Arrhenius law over this temperature range, an activation energy *E*_*AR*1_ = 7.9 ± 0.7 kJ mol^−1^ is returned, which is compatible with the energy of confined water under a reduced hydrogen-bonding condition^37^,^39^ (7.7 kJ mol^−1^). Between 250 and 200 K, *τ*_*R*_ starts to decrease, reaching a minimum at 150 K, and then increasing again when going down to 10 K, where *τ*_*R*_ assumes about the same value as at 250 K. Below 250 K, the rotational dynamics increases in spite of temperature reduction. The lowering of the rotational relaxation time below 250 K is a clear mark of the onset of a more mobile orientational regime; this behavior can only be explained with the appearance of the single-file dynamics. Since *τ*_*R*_ is larger at 200 K than at 150 K, the minimum of the rotational relaxation time should take place at 150 < *T* < 200 K, i.e., at the highest temperature compatible with the ice channel formation. Of course, due to the low number of available experimental points (mainly due to the long time required to get any point at fixed temperature), the temperature signaling the change in the orientational dynamics can be only identified indirectly (see below). Yet, our major goal was not to show the exact temperature at which the dynamic regime changes, but rather that, when temperature is lowered, the rotational diffusion of water increases to such an extent that molecules are close to behave as a quasi-free rotor.

**Figure 1:**
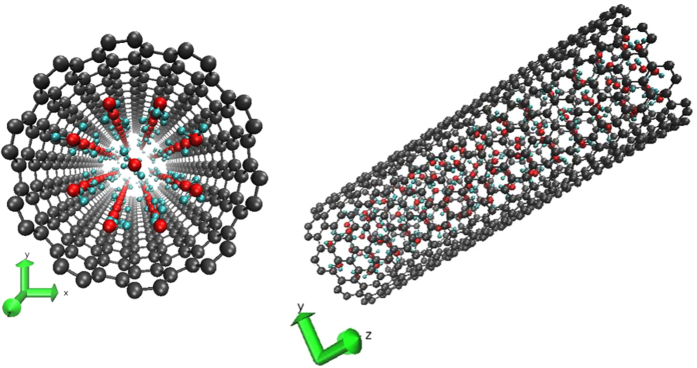
Low-temperature view of the water-filled SCWNT. The SWCNT transverse section is shown to the left side of the panel; the side view to the right. The SWCNT carbon atoms are in dark grey, the oxygen and hydrogen atoms of water molecules are in red and cyan, respectively. The single-file of water molecules is placed along the SWCNT main axis (z-axis), while the octagonal ice-channel wall is about halfway between the water single-file and the SWCNT wall.

**Figure 2:**
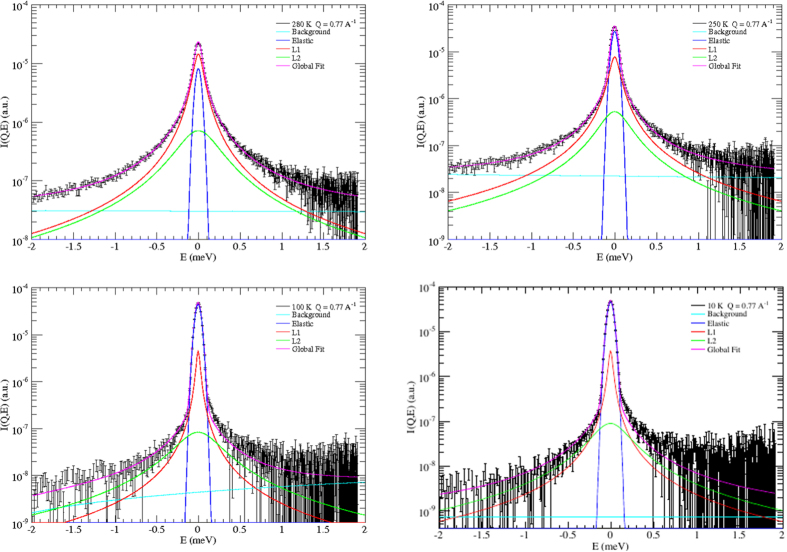
Examples of global results of data fitting to Eq. 1 **(magenta line) at 280, 250, 100, and 10 K, and at** ***Q***** = 0.77 Å**^**−1**^. The elastic peak (blue line) has the same shape of the resolution function. *L*1 (red line) refers to the translational Lorentzian term in Eq. 1 (first Lorentzian in Eq. 2). *L*2 (green line) refers to the roto-translational term (second Lorentzian in Eq. (2)). The background contribution is reported in light blue. At this *Q*-value the weight of the Bessel function 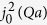 is dominant when compared to 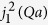, so making the translational term the leading one.

**Figure 3:**
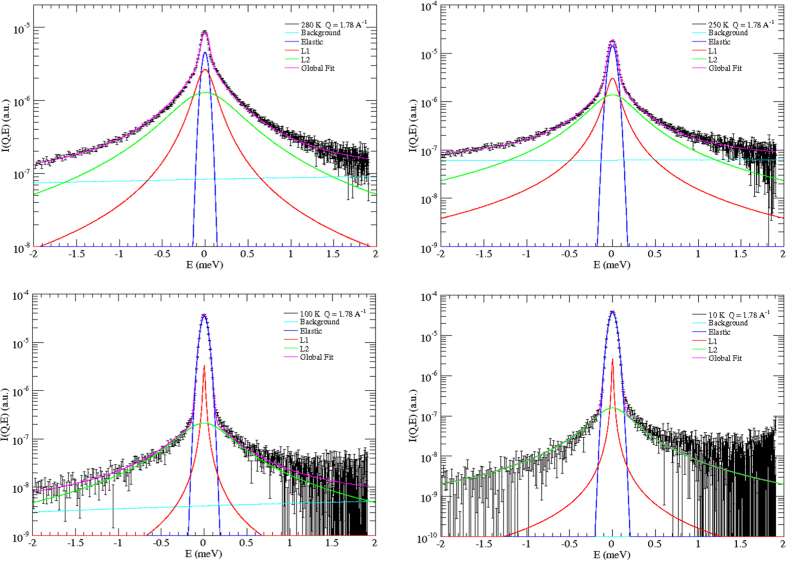
Examples of global results of data fitting to Eq. (1) (magenta line) at 280, 250, 100, and 10 K, and at *Q* = 1.78 Å^−1^. The elastic peak (blue line) has the same shape of the resolution function. *L*1 (red line) refers to the translational Lorentzian term in Eq. (1) (first Lorentzian in Eq. (2)). *L*2 (green line) refers to the roto-translational term (second Lorentzian in Eq. (2)). The background contribution is reported in light blue. At this *Q*-value the weight of the Bessel function 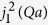 is dominant when compared to 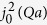, so increasing the weight of the roto-translational term with respect to Fig. 2.

The temperature dependence of the immobile proton fraction *f*_*C*_ (Fig. 5) gives an insight into the temperature value at which the orientational dynamics changes, and confirms the establishment of the single-file structure. The temperature at which *f*_*C*_ shows a net change of slope is 190 ± 2 K. Figure 5 clearly suggests that, from room temperature to about 190 K, a dramatic change in the proton mobility takes place, while below about 190 K the immobile water fraction is almost complete. From data shown in Fig. 5, it is easy to infer that, at the lowest temperatures investigated, 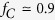, which corresponds to a fraction 8:1 between shell and single-file water molecules; therefore, the transversal section of the ice channel is compatible with an octagonal structure (Fig. 1). This confirms one of the conjectures made about the ice-channel structure^20^,^23^ for the first time. The temperature of 190 ± 2 K can be taken the reference temperature for the full formation of the ice channel^6^. Therefore, the minimum of the single-file rotational relaxation time should occur at about 190 K: this temperature value falls well inside the above-mentioned range. Of course all frozen water, whose very slow diffusion lies under the instrumental resolution, is included in the *f*_*C*_ term. Even though this is to some extent experimentally unavoidable, such a water component should be negligible because the confinement geometry forces molecules to arrange as a square-ice at a low enough temperature^6^. However, the possible formation of glassy water may only affect the ratio of immobile to mobile water per SWCNT, as it could prevent the channel formation.

**Figure 4:**
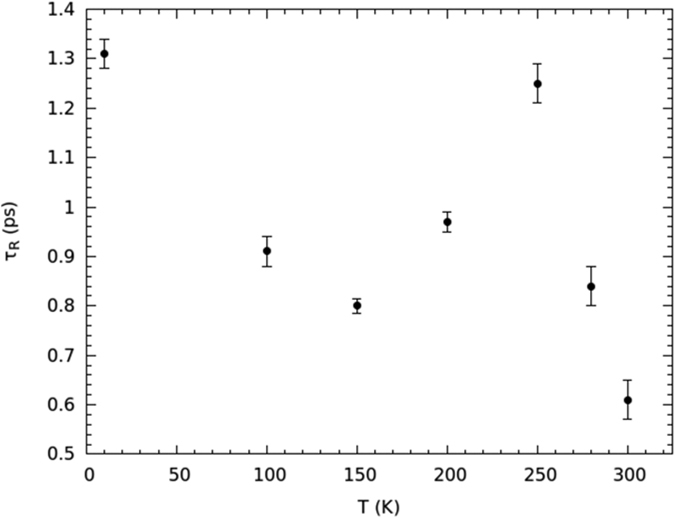
The temperature-dependent rotational relaxation time obtained by the fit to Eq. (1).

Figure 4 proves that, when the single-file of water is formed, rotational diffusion gets enhanced. Below 150 K the rotational relaxation time increases, following an energy-activation mechanism probably different from the Arrhenius model. If an Arrhenius law is tested on experimental points at 150, 100 and 10 K, an orientational activation energy of *E*_*AR*2_ = 0.25 ± 0.08 kJ mol^−1^ is found^26^. The large error confirms that the Arrhenius law no longer describes the low temperature range of rotational diffusion, probably because of quantum effects that are relevant at these temperatures, apart from the low number of experimental points. However, this marked reduction of the activation energy, though inaccurate, gives an indication that the orientational motion of water becomes dramatically enhanced in such a temperature interval.

To get some more quantitative information about how much the orientational motion is close to a water free rotor, a simple calculation can be made. Water in the gas phase behaves as an asymmetric rotor with three rotational constants 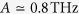, 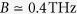, and 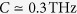. The rotation axis of the *B* constant is associated with rotations around the main molecular symmetry axis, i. e., the axis that bisects the H–O–H angle^40^. In order to compare the orientational dynamics of water molecules arranged in the single-file structure to the free rotational dynamics of a water molecule in the gas phase, the free-rotator *B* constant is the one that must be taken into account. This happens because the symmetry axis of the ice channel (Fig. 1) is very close to the rotation axis associated to the *B* constant of water molecules arranged in the single-file configuration, while rotations associated to *A* and *C* constants can be ignored, being very short-lived^41^.

Owing to the value of the *B* constant, the *J* population distribution of rotational states at 150, 100, and 10 K is very narrow, and the average rotational frequency of the free molecule is effectively representative of molecular rotation. The average rotational energy is given by 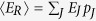, where *E*_*J*_ is the rotational energy associated to the *B* constant and *p*_*J*_ is the probability associated to the *J* state at 150, 100 and 10 K, respectively. The average free rotational frequency related to the *B* constant turns out about 2.9 THz at 150 K, 1.9 THz at 100 K, and 0.05 THz at 10 K.

Due to the confining geometry, the orientational mechanism and timing of water molecules in the single-file chain almost exclusively depend on the breaking and reformation of the hydrogen bonds within the chain, therefore on the time-scale of the bond lifetime *τ*_*HB*_. Even though explicit information on the hydrogen-bond lifetime for water in the chain configuration at 150 K and below are not available, the assumption that *τ*_*HB*_ is a few ps should be reasonable, as it is *τ*_*HB*_ ≥ 1 ps in confined environments at temperatures higher than 150 K^42^,^43^. Therefore, at 150 and 100 K, the orientational motion falls in the so-called free-rotation regime condition, i.e., 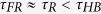, where *τ*_*FR*_ is the free rotation period. The free-rotor regime means that a molecule goes through one or more free rotation revolutions before it faces a hydrogen bond ‘collision’^44^. Under this condition the rotational relaxation time *τ*_*R*_ is a probe of molecular rotation of water in the single-file configuration. The orientational frequencies associated to *τ*_*R*_ appear to be 1.3 THz at 150 K and 1.1 THz at 100 K, respectively, which are pretty close (same order of magnitude) to the average rotational frequencies associated to the *B* constant of free molecules at the same temperatures. Conversely, at 10 K the condition 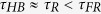 should be fulfilled, and it indicates that reorientations are limited by the lifetime scale of hydrogen bonds. In this case *τ*_*R*_ gives indications on the collisional dynamics, i.e., about the hydrogen-bond dynamics rather than molecular rotation. This would justify the great difference between the orientational frequency of 0.8 THz and the 0.05-THz average rotation frequency associated to the *B* constant of free molecules at 10 K. The conclusions drawn about the system at 150 and 100 K indicate that the rotational behavior of water molecules in the single-file arrangement is very close to the one of water molecules in the gas phase, at least for what concerns rotations associated to the *B* constant. The same conclusion can, of course, be extended to the 10-K case, unless an unlikely structural change in the ice channel or chain arrangement occurs between 100 and 10 K.

### Translational dynamics

The behavior of the translational diffusion coefficient *D*_*T*_ is reported in Fig. 6. It is similar to the one of *τ*_*R*_, even though the lowest temperature at which *D*_*T*_ is appreciable is 150 K, the error being over the 100% for colder determinations, so that a reliable quantitative assessment is not possible any longer. In the interval from 300 to 250 K an Arrhenius fit returns an *E*_*AT*1_ = 26.0 ± 6.6 kJ mol^−1^ activation energy, which is larger than the one associated to diffusion in bulk water, estimated^45^,^46^ as about 18 kJ mol^−1^. On the other hand, water under these conditions should be closer to the ‘fragile’ dynamic state^24^,^47^; and a Vogel-Fulcher-Tammann (VFT) function, rather than an Arrhenius law, should govern the temperature dependence of the translational dynamics. Because of the few experimental points available, the difference between the two behaviors cannot be appreciated, and this probably leads to an overestimation of the activation energy. Anyway, under similar confining conditions and temperatures, *D*_*T*_ values very similar to ours are found^4^,^24^.

Due to the higher activation energy compared to rotations, the low temperature range of *D*_*T*_ comprises only two experimental points, at 200 and 150 K (Fig. 6), respectively, therefore an estimate of the activation energy would give very inaccurate results^26^, also because at 200 K the ice channel is not fully formed yet. However, the *D*_*T*_-value at 200 K is larger than the one at 250 K, which is, in turn, comparable to the one at 150 K. All of that is a mark of the same mechanism already met for *τ*_*R*_(*T*), i.e., the water molecules increase their mobility after the ice-channel formation because of the reduction of the coordination number per molecule. The behavior of *D*_*T*_(*T*) fully confirms the onset of a single-file dynamics, and it is utterly coherent with the behavior of *τ*_*R*_(*T*).

The enhancement of the translational and rotational diffusion of water, in spite of temperature reduction, is a measure of water’s adjustment to the symmetry of confinement via the dispersion of the free energy made available by the reduction of hydrogen bonds per water molecule into the roto-translational degrees of freedom.

## Conclusions

Novel QENS measurements on a sample of (10, 10) SWCNTs, over an extended temperature range offer, for the first time, an evidence of the full temperature-dependent pattern of the dynamics of water confined within an axial geometry at the nanometer scale. The very special feature of this pattern is the onset of single-file dynamics, with molecules exhibiting a quasi-free rotor behavior. This special dynamic regime occurs at low temperatures, after the establishing of sub-nanometer confinement due to the ice-channel formation. This peculiar molecular arrangement allows water to show an active dynamics even at temperatures as low as 10 K. The analysis of the translational dynamics completely confirms the rotational counterpart.

## Methods

Measurements were performed at the Institut Laue Langevin (ILL, Grenoble, France) at the IN6 time-of-flight spectrometer, using an incident wavelength of 5.1 Å, with an energy resolution of about 70 *μ*eV. The momentum transfer *Q* spanned the range from 0.38 to 2.0 Å^−1^ corresponding to the angular range from 17.9° to 110.3°.

The dry SWCNT sample was prepared from high-quality anhydrous open-ended purified (10, 10) SWCNTs whose average diameter (1.4 ± 0.1 nm) was assessed by standard transmission electron microscopy and Raman measurements. Dry SWCNTs were placed in a 0.3-mm-deep annular aluminum sample holder, designed to make multi-scattering events negligible, and then further dried in an oven at 45 °C for 2 hours. Then the sample holder was sealed using an indium gasket.

The water-filled SWCNT sample was prepared by exposition of the dry sample to saturated vapor from a distilled and de-ionized water bath at about 120 °C for two hours, in an enclosed environment at a pressure of about 2 atm. The excess water was then let evaporate at 45 °C in order to reach the optimal water mass fraction. The optimal filling, in terms of the H_2_O/SWCNT mass ratio, was found to be 11.3%^6^,^24^. The whole water mass content of the sample was 0.23 g. After optimal filling was reached, an indium gasket sealed the sample holder.

For the QENS data analysis, the spectra were arranged into 11 angle-averaged groups. The data were collected along the isothermal path at each investigated temperature (namely, 300, 280, 250, 200, 150, 100, and 10 K). Analogous scans were taken for the empty cell, the dry SWCNT sample, and the vanadium standard in order to assess the background contribution, experimental resolution and detector efficiency. Since no temperature-dependent effect was observed in the dry SWCNT sample, the resolution was set starting from spectra obtained for this sample at 10 K.

The removal of the contribution from the aluminum sample holder, as well as vanadium normalization and correction, was performed by well-established instrument routines.

The experimental Gaussian-modeled resolution was used for the deconvolution of the experimental spectra from the resolution function *R(Q, E*). *f*_*C*_, Γ_*T*_(*Q*), *τ*_*R*_ and background constants were set as free parameters in the data fit to Eq. 1.

The relative weights of the two Lorentzians in Eq. 2 were just determined by the spherical Bessel functions *j*_0_(*Qa*) and *j*_1_(*Qa*), i.e., with no introduction of further free parameters. The two Lorentzians describe the sample fraction (1 − *f*_*C*_) of mobile hydrogens, where *f*_*C*_ is the immobile fraction.

The two Lorentzians in Eq. 2 are the result of the convolution between the translational Lorentzian and the *j*_0_(*Qa*) EISF (Elastic Incoherent Structure Factor) plus the *j*_1_(*Qa*) term of the Sears expansion^32^, which takes rotational diffusion into account^27^,^32^. The *j*_1_(*Qa*) term is the only non-elastic term that gives significant contribution at the *Q*-values spanned in our experiment^26^.

The rotational diffusion time was determined by averaging the values obtained at *Q* transfers ranging from 0.77 to 1.78 Å^−1^, i.e., excluding the smallest and largest *Q*s for which the rotational contribution is, respectively, too weak or similar to the translational component^26^. The rotational relaxation time *τ*_*R*_(*Q*) has been reported in Fig. 7. For the sake of clarity, to avoid the superposition of data at different temperatures, *τ*_*R*_(*Q*) has been only reported for the particularly significant temperatures that characterize the single-file dynamics. As one can see, the rotational relaxation time is quite constant within the error for almost all the *Q*-values, if the restricted range is taken into account. On the other hand, the average of *τ*_*R*_(*Q*) made on the entire *Q*-range at such temperatures does not change appreciably when compared to the restricted counterpart. The difference is more marked at higher temperature, when the average is calculated over the complete range, even though, for *Q*s ranging from 0.77 to 1.78 Å^−1^, the *τ*_*R*_(*Q*) is quite stable and independent of any change in either the fit energy-range or the background terms^26^.

**Figure 5:**
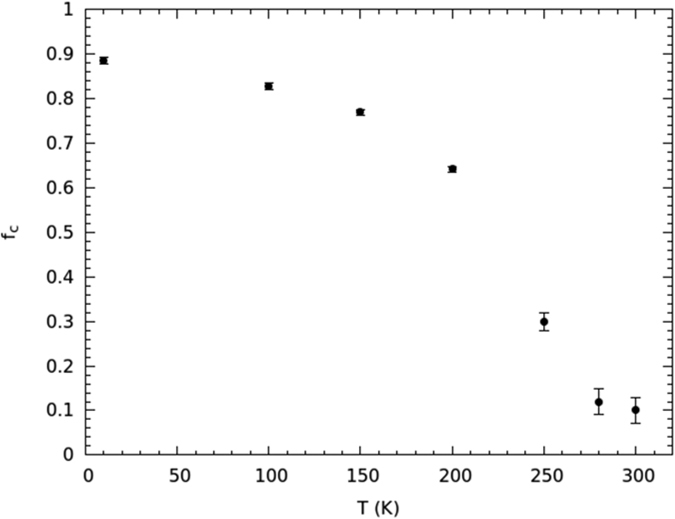
The temperature dependence of protons’ immobile fraction *f*_*C*_.

**Figure 6:**
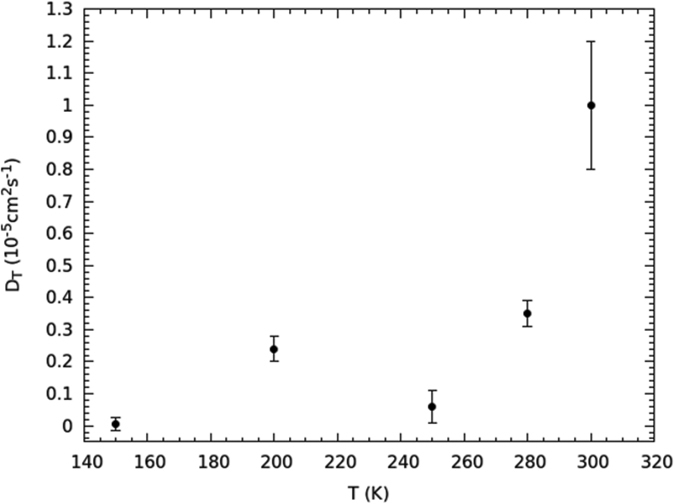
The temperature-dependent translational diffusion coefficient *D*_*T*_ obtained by the fit toEq. (1).

**Figure 7:**
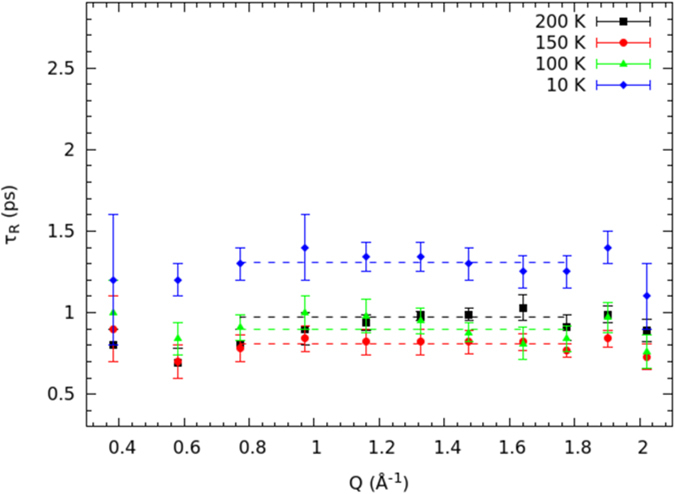
The *Q*-dependent rotational relaxation time. For the sake of clarity, only the particularly significant temperatures that characterize the single-file dynamics have been reported. The dashed lines indicate the average values obtained at *Q* ranging from 0.77 to 1.78 Å^−1^.

**Figure 8:**
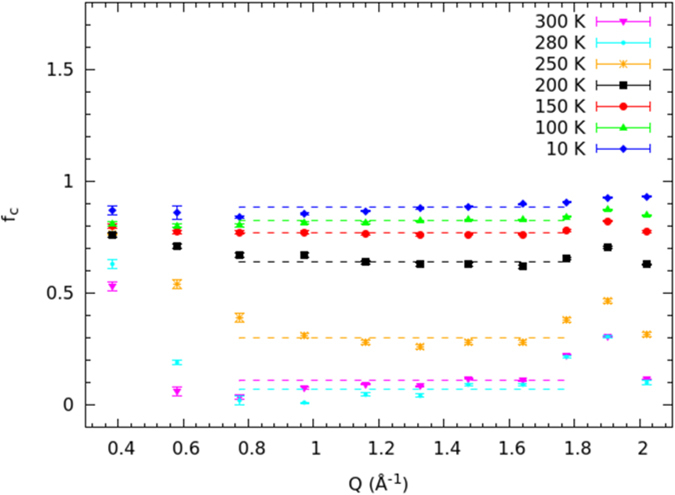
The *f*_*C*_(*Q*) distribution at the investigated temperatures as obtained from the fit toEq. (1). The dashed lines are the average values obtained at *Q*s ranging from 0.77 to 1.78 Å^−1^.

If the fraction of immobile hydrogens is interpreted as the fraction of (rigid) water molecules that are characterized by *D*_*T*_(*Q*) → 0 and *τ*_*R*_(*Q*) → ∞, *f*_*C*_(*Q*) it is almost independent of *Q*:


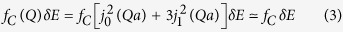


which is over-90% verified for *Qa* < 1.5, that is, over most of our *Q*-range. The *f*_*C*_(*Q*) data obtained from Eq. 1 have been reported in Fig. 8, at the various investigated temperatures. This figure shows that the *f*_*C*_(*Q*) data placed in the mid-*Q* region are quite constant at all temperatures and, for temperatures from 200 down to 10 K, they are almost constant on the whole range. This behavior in the single-file temperature region is seemingly ascribable to the condition under which the above relation works, since most of the sample is in the ice phase. For temperature higher than about 200 K, the *Q*-dependence seems instead to play a more active role at low *Q*s as well, probably because molecules with an almost fixed center of mass, but with some rotational activity, could characterize the immobile fraction at such temperatures. The *f*_*C*_(*T*) data reported in Fig. 5 have been obtained by averaging the *f*_*C*_(*Q*) values from *Q* = 0.77 Å^−1^ to *Q* = 1.78 Å^−1^.

**Figure 9:**
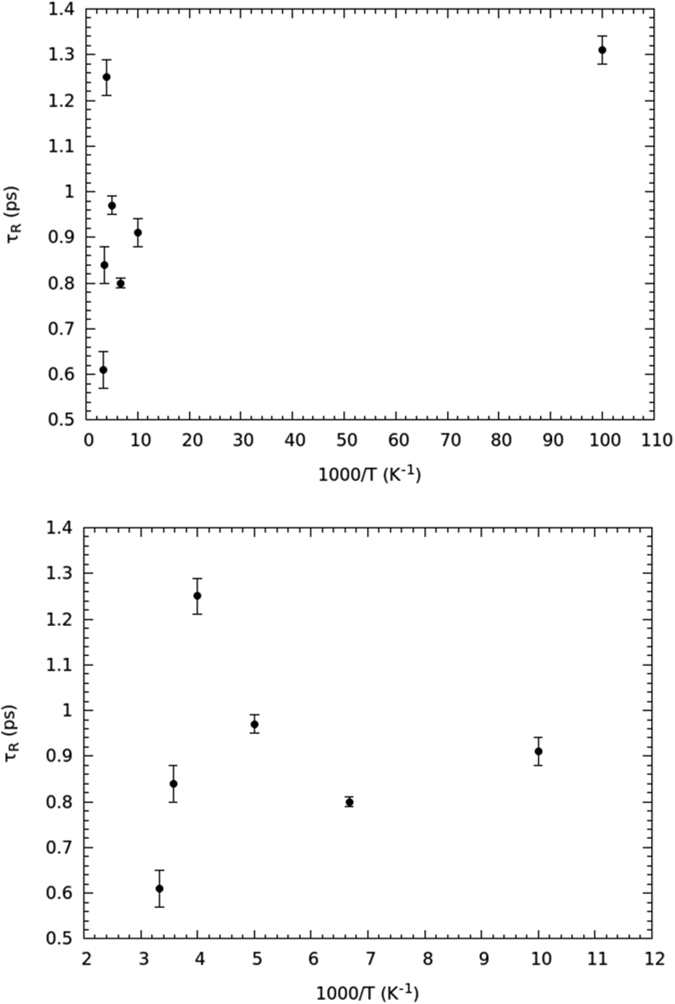
Semi-log plot of the rotational relaxation time *τ*_*R*_ vs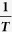. Top: the entire temperature range from room temperature to 10 K. Bottom: the zoom of the same region from room temperature to 100 K.

In Fig. 9 the semi-log plot of *τ*_*R*_ vs 1/T is reported. In the highest temperature domain, i.e., from room temperature down to 250 K, the rotational relaxation time vs 1/T shows a linear behavior (Fig. 9), within the limitations related to the low number of experimental points. The 200 K point seems to show a behavior intermediate between the high temperature regime and the single-file regime, probably due to the incomplete formation of the ice channel. The full single-file temperature range for orientational dynamics, which extends from 150 K down to 10 K, seems to suggest a non-Arrhenius behavior (Fig. 9). However, the reduction of the energy barrier for rotational diffusion is evident due to the small jump in the *τ*_*R*_ values with respect to the large temperature interval.

**Figure 10:**
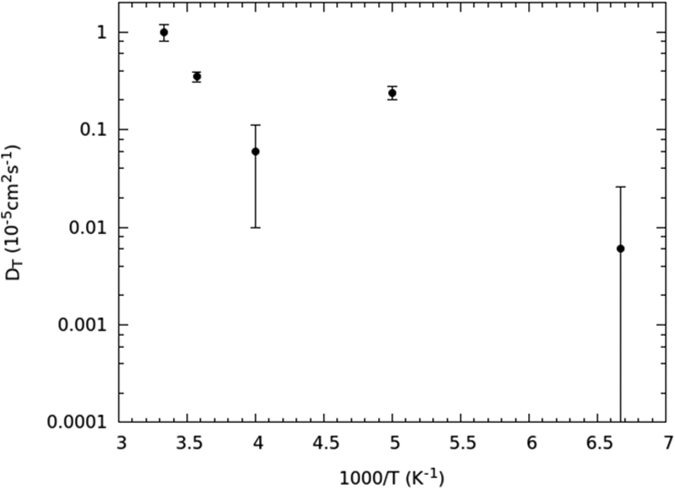
Semi-log plot of the translational diffusion coefficient *D*_*T*_ vs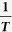.

The semi-log plot of the translational diffusion constant *D*_*T*_ is reported in Fig. 10. Also in this case the behavior of the first three experimental points, that is, for temperature between 300 and 250 K, is compatible with a linear trend, even though with an error greater than the one associated to *τ*_*R*_(*T*^−1^). This low number of experimental points produces an inaccuracy that does not allow discriminating an Arrhenius law from a VFT function. This may cause an overestimation of the activation energy. For what concerns the single-file temperature regime, here the uncertainty is too high to get some quantitative indication about the activation energy, as only two experimental points with large errors are available.

## Additional Information

**How to cite this article:** Briganti, G. *et al*. Neutron scattering observation of quasi-free rotations of water confined in carbon nanotubes. *Sci. Rep.* **7**, 45021; doi: 10.1038/srep45021 (2017).

**Publisher's note:** Springer Nature remains neutral with regard to jurisdictional claims in published maps and institutional affiliations.
